# Time trends in adherence to UK dietary recommendations and associated sociodemographic inequalities, 1986-2012: a repeated cross-sectional analysis

**DOI:** 10.1038/s41430-018-0347-z

**Published:** 2018-11-16

**Authors:** Amy Yau, Jean Adams, Pablo Monsivais

**Affiliations:** 1 Centre for Diet and Activity Research, MRC Epidemiology Unit, University of Cambridge, Cambridge, United Kingdom; 2 Department of Nutrition and Exercise Physiology, Elson S Floyd College of Medicine, Washington State University, Spokane, Washington United States

**Keywords:** Epidemiology, Risk factors, Epidemiology, Health policy

## Abstract

****Background/objectives**:**

Little is known about time trends in diet quality and associated inequalities in the UK. This study aimed to examine trends in adherence to four UK dietary recommendations, overall and among sociodemographic subgroups, from 1986 to 2012.

****Subjects/methods**:**

We conducted a repeated cross-sectional analysis using data from three UK diet surveys: Dietary and Nutritional Survey of British Adults 1986–87 (*n* = 2018), National Diet and Nutrition Survey (NDNS) 2000–01 (*n* = 1683) and NDNS Rolling Programme 2008–12 (*n* = 1632). We measured adherence to dietary recommendations for fruit and vegetables, salt, oily fish, and red and processed meat, estimated using food diary record data. We compared adherence across surveys and by four sociodemographic characteristics: sex, age, socioeconomic position and ethnicity.

****Results**:**

Overall, population adherence to dietary recommendations was low to moderate, but improved over time. There were inequalities in adherence to all recommendations at all timepoints according to one or more sociodemographic characteristic. When inequalities were present, women, older adults, those with non-manual occupations and non-Whites were more likely to adhere to dietary recommendations. Although some dietary inequalities declined, most persisted across the three surveys.

****Conclusions**:**

The persistence of most inequalities highlights the need for further interventions to reduce dietary inequalities as well as improve overall population diet. The greatest simultaneous improvement in population adherence and reduction of inequalities was observed for salt, which may reflect the success of the UK Salt Reduction Programme. Similarly comprehensive programmes should be encouraged for other dietary components.

## Introduction

Dietary factors account for nearly one in five deaths and are the second leading risk factor for global disability [1]. In England, consumption of unhealthy diets is the biggest behavioural risk factor for morbidity and mortality, accounting for 10.8% of Disability-Adjusted Life Years lost in 2013 [2]. Current nutrition surveillance data from the United Kingdom suggest that dietary recommendations are largely not met by the population [3]. It has been estimated that if the UK population met current dietary recommendations, approximately 30,000 deaths per year could be prevented, 15,000 and 7500 of which would be a result of meeting the fruit and vegetable recommendation and salt recommendation, respectively [4]. Health benefits would also be seen by complying with recommendations for oily fish, and red and processed meat: higher fish intake, especially oily fish, is associated with lower incident rates of cardiovascular disease [5], and lower red and processed meat consumption with reduced mortality from cardiovascular disease and cancer [6].

Alongside suboptimal population diet quality, dietary risk factors are not distributed equally across population subgroups leading to dietary inequalities. Although inequalities in diet have been documented cross-sectionally for over 80 years [7], little is known about the evolution of dietary inequalities seen today. Studies conducted in the United States and the Netherlands found persisting or widening inequalities in diet quality by education, income, ethnicity, age and sex [8–12]. In the United Kingdom, most research has focused specifically on socioeconomic inequalities and a small number of food groups, reporting persisting gaps in fruit and vegetable intake and intake of high-fat and high-sugar foods [13–16]. Thus, little is known about other sociodemographic inequalities in the consumption of a wider range of food groups. In this study we aimed to examine trends in adherence to four dietary recommendations in the UK from 1986 to 2012, overall and among sociodemographic subgroups.

## Methods

### **Data sources**

We used data from three national diet surveys to conduct a repeated cross-sectional analysis: Dietary and Nutritional Survey of British Adults (DNSBA) 1986–87 [17], National Diet and Nutrition Survey (NDNS) 2000–01 [18] and NDNS Rolling Programme 2008–12 [19]. A rolling programme was introduced in 2008 to replace the one-off surveys previously conducted. In order to achieve a sample size comparable to previous surveys, we used data from the first four years of the Rolling Programme. All surveys used multistage random sampling and recruited a cross-section of the UK adult population. Response rates for the surveys have been reported as 70%, 47% and 58% for DNSBA 1986–87, NDNS 2000–01 and NDNS 2008–12, respectively. Full details on the survey methods and response rates are described elsewhere: DNSBA 1986–87 [17], NDNS 2000–01 [20] and NDNS Rolling Programme (2008–12) [3].

For DNSBA, ethics approval was obtained from the British Medical Association. For NDNS, ethics approval was obtained from the Oxfordshire A Research Ethics Committee. Written informed consent was obtained from all participants.

### **Inclusionand exclusioncriteria**

Respondents aged 19–64 years with sufficient dietary data (seven days of food diary records for DNSBA 1986–87 and NDNS 2000–01, and three or four days of food diary records for NDNS Rolling Programme 2008–12) were included. A small number of respondents were excluded due to insufficient information for assignment of socioeconomic position (SEP) (*n* = 29, 41 and 23 in 1986–87, 2000–01 and 2008–12, respectively).

### **Sociodemographic characteristics**

We examined adherence to dietary recommendations by four sociodemographic characteristics: sex (men and women), age (19–40 and 41–64 years), SEP (non-manual and manual occupations) and ethnicity (Whites and non-Whites). SEP was based on the occupation of the household reference person/head of house. In DNSBA 1986–87 and NDNS 2000–01, occupational social class was classified using the Registrar General’s Social Class (RGSC). The National Statistics Socioeconomic Classification (NS-SEC) replaced RGSC as the UK government’s preferred measure of occupation social class in 2001 and this was used in the NDNS Rolling Programme. For comparability, we derived the household reference person’s RGSC for respondents in the Rolling Programme using the Standard Occupational Classification 2000 and employment status [21]. Where this was not possible from the information available, we estimated RGSC from the NS-SEC category (for details see Supplementary Figure S1) [22]. Respondents were stratified into two categories for analysis: non-manual occupations (I Professional; II Managerial/Technical; III-NM Skilled Non-Manual) and manual occupations (III-M Skilled Manual; IV Partly Skilled; V Unskilled).

### **Measuring adherence to dietary recommendations**

Dietary data were collected using food diary records, weighed 7-day diaries in the first two surveys and unweighed 4-day diaries in NDNS 2008–12. We used average person-level daily intake estimates to measure adherence to the current UK recommendations for four key dietary components related to chronic diseases: fruit and vegetables ( ≥ 400 g/day), oily fish ( ≥ 140 g/week), salt ( ≤ 6 g/day), and red and processed meat ( ≤ 80 g/day). The daily average intake was multiplied by seven for the oily fish recommendation, which is expressed per week.

### **Statistical methods**

Adjusted logistic regression models were used to estimate the odds ratios (ORs), with 95% confidence intervals (CIs), for meeting the dietary recommendations by sex, age, SEP, ethnicity and timepoint, with each analysis mutually adjusted for the other variables. We examined interaction terms between the four sociodemographic characteristics and timepoint to determine whether the differences in adherence between sociodemographic subgroups changed over time. We used likelihood-ratio tests to compare models with and without interaction terms (sociodemographic characteristic × timepoint), in order to test the significance of each interaction. We also used an adjusted multiple logistic regression model to estimate the relative risk of achieving any number of these recommendations across the surveys. Significance levels were set at a two-tailed *P*-value ≤ 0.05 for all tests. All statistical analyses were performed using Stata/SE 13.

### **Sensitivity analyses**

Although all three surveys aimed to achieve population representative samples, variations in response across population subgroups can lead to non-response bias. Survey weights were provided in the second and third surveys to reduce the effects of this. In sensitivity analyses, we ran models using survey weights in the second and third surveys. This did not alter our conclusions (see Supplementary Table S1-S2). Hence, for consistency, we present all our results without survey weights.

## Results

### **Population characteristics**

Overall, 5333 individuals were included in the analyses. The proportion of respondents who were women, aged 41–64 years, in non-manual households and non-White increased over time (see Table 1).

**Table 1:** Descriptive characteristics of study population

	1986–1987 (*n* = 2018)	2000–2001 (*n* = 1683)	2008–2012 (*n* = 1632)	Total (*n* = 5333)
Sex, *n* (%)				
Men	991 (49.1)	753 (44.7)	705 (43.2)	2449 (45.9)
Women	1027 (50.9)	930 (55.3)	927 (56.8)	2884 (54.1)
Age, years, *n* (%)				
19–40	1055 (52.3)	794 (47.2)	720 (44.1)	2569 (48.2)
41–64	963 (47.7)	889 (52.8)	912 (55.9)	2764 (51.8)
Socioeconomic position^*^, *n* (%)				
Non-manual	973 (48.2)	970 (57.6)	987 (60.5)	2930 (54.9)
Manual	1045 (51.8)	713 (42.4)	645 (39.5)	2403 (45.1)
Ethnicity, *n* (%)				
White	1940 (96.1)	1593 (94.7)	1473 (90.3)	5006 (93.9)
Non-White	78 (3.9)	90 (5.4)	159 (9.7)	327 (6.1)

Non-manual = professional (I), managerial/technical (II) and skilled non-manual (III-NM). Manual = skilled manual (III-M), partly skilled (IV) and unskilled (V).

^*^Based on RGSC classification

### **Adherence to dietary recommendations**

Table 2 shows the proportion of respondents meeting each dietary recommendation over time and the adjusted OR for achieving each recommendation compared with the previous survey. In 2008–12, over 60% of respondents achieved the salt recommendation, under half achieved the red and processed meat recommendation, and around 20% achieved the recommendations for fruit and vegetables or oily fish. The odds of meeting each recommendation increased over time, except for red and processed meat between 2000–01 and 2008–12, where there was no significant change. The greatest change in adherence was seen for the salt recommendation between 2000–01 and 2008–12: OR 2.63 (95% CI 2.26, 3.08). Table 2 also shows the proportion of respondents meeting any number of these recommendations and the relative risk ratio for doing so between surveys. The proportion of respondents adhering to multiple dietary recommendations was low, but increased over time.

**Table 2:** Changes in adherence to dietary recommendations over time

	1986–1987 (*n* = 2018)	2000–2001 (*n* = 1683)	2008–2012 (*n* = 1632)	2000–01 vs. 1986–87	2008–12 vs. 2000–01
Adherence to individual dietary recommendations, *n* (%)				OR (95% CI) of meeting recommendation	
Fruit and vegetables	168 (8.3)	271 (16.1)	341 (20.9)	1.97 (1.60 to 2.42)	1.32 (1.10 to 1.58)
Salt	690 (34.2)	682 (40.5)	1002 (61.4)	1.27 (1.09 to 1.47)	2.63 (2.26 to 3.08)
Oily fish	171 (8.5)	250 (14.9)	303 (18.6)	1.78 (1.45 to 2.20)	1.28 (1.06 to 1.54)
Red and processed meat	602 (29.8)	739 (43.9)	689 (42.2)	1.77 (1.54 to 2.04)	0.88 (0.76 to 1.02)
Number of dietary recommendations adherent to, *n* (%)				RRR (95% CI) of meeting recommendations	
0	892 (44.2)	511 (30.4)	318 (19.5)	0.69 (0.59 to 0.82)	0.62 (0.51 to 0.75)
1	682 (33.8)	565 (33.6)	562 (32.2)	REF	REF
2	388 (19.2)	469 (27.9)	469 (27.9)	1.46 (1.22 to 1.74)	1.13 (0.95 to 1.35)
3	51 (2.5)	113 (6.7)	185 (11.3)	2.55 (1.79 to 3.62)	1.60 (1.23 to 2.09)
4	5 (0.23)	25 (1.5)	42 (2.6)	5.66 (2.15 to 14.91)	1.72 (1.03 to 2.88)

*CI* confidence interval, *OR* odds ratio, *RRR* relative risk ratio

ORs and RRRs are adjusted for sex, age, socioeconomic position and ethnicity

### **Sociodemographic inequalities in meeting dietary recommendations**

**Fig. 1:**
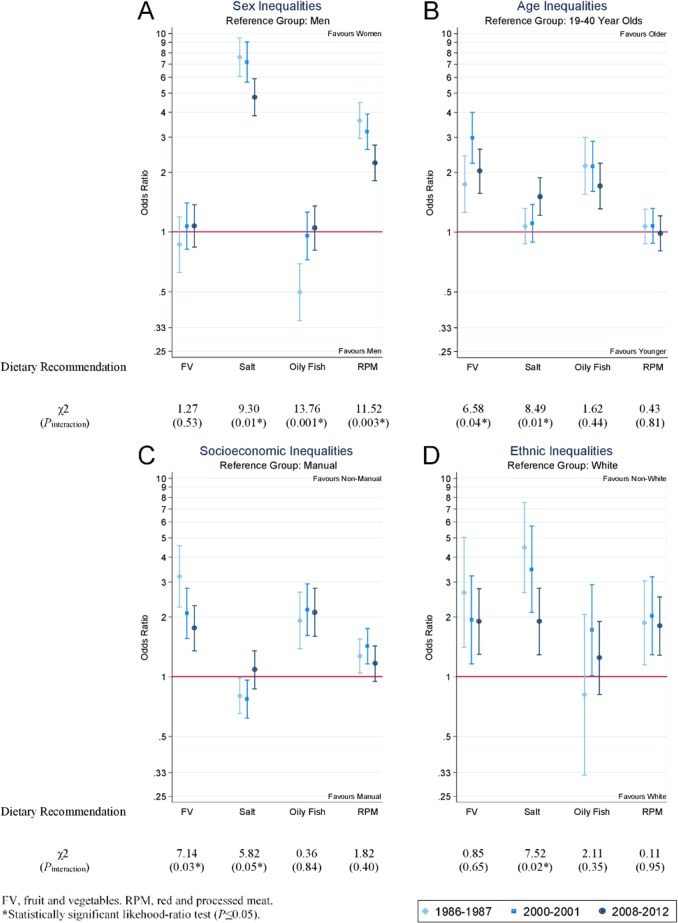
Adjusted odds ratios (95% CIs) for adhering to dietary recommendations by sociodemographic characteristics, 1986-2012. **a** Sex inequalities (reference group: men). **b** Age inequalities (reference group: 19–40 year olds). **c** Socioeconomic inequalities (reference group: manual occupations). **d** Ethnic inequalities (reference group: White). All odds ratios (95% CIs) are mutually adjusted for the other sociodemographic characteristics studied

Figure 1 shows the adjusted ORs (95% CI) for meeting the four dietary recommendations by sociodemographic characteristic. We also present the results of likelihood-ratio tests used to test for interactions between the sociodemographic characteristics and timepoint, and thus changes in sociodemographic inequalities over time.

#### Sex inequality in meeting dietary recommendations

There was no sex inequality in achieving the fruit and vegetable recommendation at any time. However, women were more likely than men to adhere to the salt, and red and processed meat recommendations at all timepoints. The magnitude of these inequalities reduced over time (*P* = 0.01 and 0.003, respectively). Men were more likely to adhere to the oily fish recommendation than women in 1986–87, but this inequality was not observed in later surveys (*P* = 0.001). Further details are shown in Supplementary Table S3.

#### Age inequality in meeting dietary recommendations

Age inequality in adherence to the fruit and vegetable recommendation was observed in all three surveys, with older adults more likely to adhere than younger adults. The magnitude of this inequality fluctuated over time: getting wider in 2000–01, then narrower in 2008–12 (*P* = 0.04). Age inequality in meeting the salt recommendation emerged between the second two surveys, favouring the older group (*P* = 0.01). The older group was more likely to meet the oily fish recommendation than the younger group. This relationship persisted without significant change across the three surveys (*P* = 0.44). There was no age inequality in adherence to the red and processed meat recommendation at any point. Further details are presented in Supplementary Table S4.

#### Socioeconomic inequality in meeting dietary recommendations

Socioeconomic inequality in meeting the fruit and vegetable recommendation persisted, favouring the higher socioeconomic group, but declined in magnitude over time (*P* = 0.03). There was marginal socioeconomic inequality in meeting the salt recommendation in the first two surveys, which favoured the manual group. This difference did not persist to the last survey (*P* = 0.05). Socioeconomic inequality in adherence to the oily fish recommendation, favouring the higher socioeconomic group, was observed at all three timepoints without evidence of significant change (*P* = 0.84). There was marginal-to-no evidence of socioeconomic inequality in adherence to the red and processed meat recommendation at all timepoints. More information is presented in Supplementary Table S5.

#### Ethnic inequality in meeting dietary recommendations

Non-Whites had higher odds of meeting all dietary recommendations than Whites, except for oily fish. These inequalities persisted across all three surveys, with only ethnic inequality in adherence to the salt recommendation reducing (*P* = 0.02). More information is available in Supplementary Table S6.

## Discussion

This is one of the first studies to investigate trends in dietary inequalities by multiple sociodemographic characteristics. Furthermore, this is the first study to do so by looking at adherence to multiple dietary recommendations in the United Kingdom. We found that most dietary inequalities identified in 1986–87 persisted in 2008–12. Although some inequalities reduced in magnitude over the study period, only sex inequality in meeting the oily fish recommendation was extinguished. Overall, adherence to dietary recommendations was low to moderate, but improved over time. The proportion of respondents meeting multiple recommendations also increased with time.

### **Strengths and limitations of this study**

We used data from three national diet surveys with similar methodologies, allowing comparison over a 26-year period. Throughout, food diaries were used to collect dietary data—one of the most accurate methods of dietary assessment at the population level [23]. However, similar to all self-reported methods of dietary assessment, diaries may be subject to social desirability bias. The switch from 7-day weighed diaries to 4-day unweighed diaries in 2008–12 may have also introduced time-varying bias. We combined four years of data from the NDNS Rolling Programme in our last timepoint to achieve a sufficient sample size for subgroup analyses. Although more recent years of data from the Rolling Programme are now available, we excluded these in order to minimise any within-timepoint variations.

Across the three surveys, non-disaggregated data were used to obtain dietary intake estimates. Mixed dishes were coded by their meat/fish component. For example, 400 g of lamb stew, consisting of 300 g of lamb and 100 g of vegetables, would be coded as a lamb dish and all 400 g would contribute to the estimated intake of red and processed meat, but not fruit and vegetable intake. Consequently, we likely overestimated oily fish, and red and processed meat intake, and underestimated fruit and vegetable intake in all surveys. More accurate estimates where mixed dishes are disaggregated into their ingredients were available for the NDNS Rolling Programme [24], but not for earlier surveys. To assess the implications for our study, we compared adherence to dietary recommendations using estimated intake of these food groups from disaggregated and non-disaggregated data in the NDNS Rolling Programme (see Supplementary Table S7). Overall adherence was 10% higher for fruit and vegetables, 2% lower for oily fish and 20% higher for red and processed meat, when using disaggregated estimates compared with non-disaggregated estimates. The inequalities observed were similar for the fruit and vegetable and oily fish recommendations for both methods of intake estimation. However, sex and socioeconomic inequalities in adherence to the red and processed meat recommendation were magnified when based on disaggregated estimates. An increased reliance on ready meals could mean that consumption of mixed dishes has increased over time [25], affecting the accuracy of non-disaggregated estimates more in later surveys compared to earlier surveys. We were unable to test the effect of disaggregation over time in our study, but if true, the general trend of modest improvement we observed in overall adherence is likely underestimated, whereas the reduction in sex inequality we reported for adherence to the red and processed meat recommendation may be overestimated.

In all three surveys, salt intake was consistently estimated using a nutrient databank. This was first developed for DNSBA 198–87, and subsequently updated for NDNS [26]. These estimates do not include discretionary salt added at the table or during cooking. We did not use the more accurate estimates from urinary sodium due to the small sample sizes. In NDNS 2000–01, dietary estimates of salt intake were 20% lower than urinary estimates [18], but underestimation was consistent across population subgroups [27].

We assessed adherence to four dietary recommendations, which are important to population health, prominent in public messaging and have quantifiable recommendations in the United Kingdom [28]. This provides good insight into diet quality using measurable benchmarks, but does not provide a comprehensive measure of diet quality. We excluded some dietary recommendations, such as sugar and fibre, due to limited data availability or a lack of comparability across the surveys. Other food groups of public health concern, such as sugary drinks, were excluded as there are currently no clear UK recommendations.

Survey weights were not available for DNSBA 1986–87. However, applying survey weights for NDNS 2000–01 and NDNS 2008–12 did not alter our conclusions (see Supplementary Table S1-S2). As such, it is likely that our results are generalisable to the United Kingdom as a whole. Moreover, our analyses focus on relative inequalities, which can be observed regardless of whether subgroups are population representative.

### **Comparison of results to other studies**

Similar to our study, persistent or widening sociodemographic inequalities in diet and modest improvements in overall population diet quality were observed in the United States and the Netherlands [8–12]. Our study was mostly consistent with other UK studies, which generally found persisting, if reducing, age and socioeconomic inequalities over time [15, 29]. However, one study found socioeconomic inequality in salt intake in the NDNS Rolling Programme (2008–11), which was inconsistent with our findings [30]. This difference could be because we used averages across the four years instead of looking at trends across each year. In addition, we used RGSC to measure SEP, rather than NS-SEC.

### **Interpretation of findings and implications for policy**

It is clear that interventions that simultaneously reduce dietary inequalities and improve overall adherence to dietary recommendations are needed. Diet quality reflects the accessibility, availability and cost of food, as well one’s food preferences, nutritional knowledge and sociocultural norms [31, 32]. These are all likely to have a role in the overall poor adherence to dietary recommendations we found. The differential effects of many of these factors across population subgroups may also be responsible for the inequalities we documented [33]. Identifying the most important determinants of both diet overall and inequalities in diet, and how to address them, is important for minimising diet-related diseases.

Cost is likely to be an important factor driving socioeconomic inequalities in diet and limiting their reduction in the United Kingdom and elsewhere. We found that socioeconomic inequalities persisted in adherence to the fruit and vegetable recommendation and oily fish recommendation. This could be due to the higher costs of diets that met these recommendations, 17% and 16%, respectively, compared with diets that did not [34]. Analysis of national UK food prices found that in absolute terms, the cost of healthier foods increased to a greater extent over a 10-year period than less healthy foods [35]. Nonetheless, food prices overall have fallen in real terms over our study period and this could have contributed to the improvement in overall adherence to dietary recommendations we observed [36]. A smaller improvement was seen between 2000–01 and 2008–12, which could be associated with the rise of food prices again between 2007 and 2012 [36].

The persisting and emerging age inequalities we found suggest that cross-sectional age differences in diet reported elsewhere are likely true age effects rather than cohort effects. Older adults are often found to have healthier diets than younger adults. Many of the barriers to healthy eating in young adults point to the food environment, social norms and pressures, and lack of skill and motivation to prepare healthy foods [33, 37, 38]. Self-reported prevalence of some of these barriers are lower in older age groups [37, 38].

Women are thought to have healthier diets, because they tend to be more health-conscious [33]. Nonetheless, we found that sex differences in diet diminished over time. This increased equality in diet quality could be a reflection of increased gender equality in society as a whole [39]. Conversely, with more women participating in the workforce and decreasing time available for household duties over time [40, 41], decreasing inequalities may be a result of women’s diets deteriorating rather than men’s improving. Indeed, we found evidence that the proportion of women adhering to the red and processed meat recommendation decreased between 2000–01 and 2008–12. Although greater gender dietary equality should be encouraged, this should not be at the expense of women’s diets. The same deterioration was seen in the non-manual group at the same time. This could also point to changes in time allocation. For example, time spent eating away from the home has increased over time, especially in the higher socioeconomic groups, and out-of-home eating is associated with lower diet quality [42, 43].

Ethnic differences in diet are often difficult to study due to the small proportion of ethnic minority individuals participating in surveys. However, we found that non-Whites had consistently higher odds of achieving dietary recommendations than Whites. This could be due to a range of factors, including different sociocultural environments and food beliefs [32]. Further focus on ethnic minorities in the United Kingdom may help to identify healthy dietary behaviours that could be promoted to the whole population.

We found reduced inequalities in adherence to the salt recommendation by sex and ethnicity over time, and a substantial increase in overall adherence between 2000–01 and 2008–12. This could be due to the UK Salt Reduction Programme introduced in 2003, which included voluntary reformulation targets for the food industry as well as public information campaigns [44, 45]. Previous studies suggest that the combination of behavioural and structural elements of this programme led to its success in reducing inequalities [46]. In contrast, a lack of such coordinated effort for other components of diet may explain persisting inequalities. An evaluation of the UK’s 5-a-day public information campaign, which aims to increase fruit and vegetable consumption, found small improvements in overall intake and inequality reduction two years following its introduction [47]. This suggests that public awareness alone is not enough to improve population diet quality substantially. The comprehensive multi-component programmes for sugar and calorie reduction recently announced in England should, therefore, be welcomed from an equity point of view [48, 49].

### **Conclusions**

We found that most sociodemographic inequalities in adherence to key UK dietary recommendations persisted between 1986 and 2012. Alongside, we found low-to-moderate, but improving, overall adherence to dietary recommendations. Further interventions to reduce dietary inequalities in the United Kingdom as well as improve overall population diet quality are needed.

## Supplementary information

Supplementary Material Legends

Supplementary Figure S1

Supplementary Table S1

Supplementary Table S2

Supplementary Table S3

Supplementary Table S4

Supplementary Table S5

Supplementary Table S6

Supplementary Table S7
