# Supplementary material for: Time trends in adherence to UK dietary recommendations and associated sociodemographic inequalities, 1986-2012: a repeated cross-sectional analysis
Source: Eur J Clin Nutr. 2018 Nov 16;73(7):997–1005. doi: 10.1038/s41430-018-0347-z (PMC6398578; doi:10.1038/s41430-018-0347-z)
Supplement: Supplementary file 1 — Supplementary Material Legends [file 41430_2018_347_MOESM1_ESM.docx]

**SUPPLEMENTARY MATERIAL**

**Supplementary Figure S1.** Flowchart for Registrar General’s Social Class estimation for NDNS Rolling Programme respondents. (.docx)

**Supplementary Table S1.** Weighted vs unweighted data: adjusted odds ratios (95% CIs) for adhering to dietary recommendations by sociodemographic characteristics. (.docx)

**Supplementary Table S2.** Weighted vs unweighted data: adjusted odds ratios (95% CIs) for adhering to dietary recommendations over time. (.docx)

**Supplementary Table S3.** Sex inequalities: *n* (%) adhering to dietary recommendations and adjusted odds ratios (95% CIs) for adherence. (.docx)

**Supplementary Table S4.** Age inequalities: *n* (%) adhering to dietary recommendations and adjusted odds ratios (95% CIs) for adherence. (.docx)

**Supplementary Table S5.** Socioeconomic inequalities: *n* (%) adhering to dietary recommendations and adjusted odds ratios (95% CIs) for adherence. (.docx)

**Supplementary Table S6.** Ethnic inequalities: *n* (%) adhering to dietary recommendations and adjusted odds ratios (95% CIs) for adherence. (.docx)

**Supplementary Table S7.** Sociodemographic inequalities in meeting dietary recommendations for fruit and vegetables, oily fish and red and processed meat, comparing non-disaggregated and disaggregated data from NDNS Rolling Programme (2008-2012). (.docx)
