# Supplementary material for: Time trends in adherence to UK dietary recommendations and associated sociodemographic inequalities, 1986-2012: a repeated cross-sectional analysis
Source: Eur J Clin Nutr. 2018 Nov 16;73(7):997–1005. doi: 10.1038/s41430-018-0347-z (PMC6398578; doi:10.1038/s41430-018-0347-z)
Supplement: Supplementary file 2 — Supplementary Figure S1 [file 41430_2018_347_MOESM2_ESM.docx]

**Supplementary Figure 1.** Flowchart for Registrar General’s Social Class estimation for NDNS Rolling Programme respondents.

1655 respondents

Classified using SOC2000

141 respondents

1514 respondents

Classified using NS-SEC8

81 respondents

60 respondents

23 respondents excluded

37 respondents

Classified using NS-SEC8 in combination with SIC2007B

**TOTAL**

1632 respondents included

There were 1655 respondents aged 19 to 64 years with 3 or 4 days of food diary records in 2008-12. Using information on the household reference person’s employment status (whether s/he was self-employed or an employee, and whether s/he was working in an organisation with more or fewer than 25 people) and the Standard Occupational Classification (SOC) 2000, we were able to classify 1514 (91.5%) of respondents into the two Registrar General’s Social Class (RGSC) categories: non-manual occupations and manual occupations. Of the remaining 141 respondents, 81 (4.9% of the original sample) were classified using their NS-SEC classification. This NS-SEC to RGSC estimation was based on the conversion table from National Statistic’s documentation for ‘continuity issues: SC, SEG and NS-SEC’^22^. A further 37 respondents (2.2%) were classified using NS-SEC combined with Standard Industrial Classification (SIC) 2007, which gave further details on the industry in which the household reference person worked. This resulted in 1632 (98.6%) respondents with estimated RGSC classifications, allowing comparison across surveys. We were unable to classify 8 (0.5%) respondents due to uncertainty regarding occupation. A further 15 respondents were excluded due to incomplete occupational data.
