# Supplementary material for: Time trends in adherence to UK dietary recommendations and associated sociodemographic inequalities, 1986-2012: a repeated cross-sectional analysis
Source: Eur J Clin Nutr. 2018 Nov 16;73(7):997–1005. doi: 10.1038/s41430-018-0347-z (PMC6398578; doi:10.1038/s41430-018-0347-z)
Supplement: Supplementary file 3 — Supplementary Table S1 [file 41430_2018_347_MOESM3_ESM.docx]

**Supplementary Table S1.** Weighted vs unweighted data: adjusted odds ratios (95% CIs) for adhering to dietary recommendations by sociodemographic characteristics.

|  | **1986-1987**  **(unweighted)**  OR (95% CI) | **2000-2001 (unweighted)**  OR (95% CI) | **2000-2001 (weighted)**  OR (95% CI) | **2008-2012 (unweighted)**  OR (95% CI) | **2008-2012 (weighted)**  OR (95% CI) |
| --- | --- | --- | --- | --- | --- |
| **Sex (reference group: men)** | | | | | |
| FV | 0.86  (0.63, 1.19) | 1.07  (0.82, 1.40) | 1.08  (0.81, 1.44) | 1.07  (0.84, 1.37) | 0.97  (0.74, 1.27) |
| Salt | 7.59  (6.07, 9.48) | 7.18  (5.69, 9.07) | 7.79  (6.02, 10.09) | 4.76  (3.83, 5.91) | 4.33  (3.39, 5.52) |
| Oily fish | 0.50  (0.36, 0.69) | 0.95  (0.73, 1.26) | 1.01  (0.75, 1.36) | 1.05  (0.81, 1.35) | 1.09  (0.82, 1.46) |
| RPM | 3.63  (2.95, 4.47) | 3.20  (2.60, 3.93) | 3.58  (2.86, 4.50) | 2.22  (1.81, 2.73) | 2.08  (1.65, 2.62) |
| **Age (reference group: 19-40)** | | | | | |
| FV | 1.74  (1.26, 2.42) | 2.98  (2.22, 4.00) | 3.14  (2.30, 4.29) | 2.02  (1.56, 2.62) | 1.95  (1.46, 2.62) |
| Salt | 1.07  (0.87, 1.31) | 1.11  (0.89, 1.38) | 1.10  (0.87, 1.40) | 1.51  (1.21, 1.88) | 1.60  (1.26, 2.03) |
| Oily fish | 2.15  (1.55, 3.00) | 2.15  (1.61, 2.87) | 2.54  (1.86, 3.47) | 1.71  (1.31, 2.23) | 1.60  (1.19, 2.16) |
| RPM | 1.07  (0.87, 1.30) | 1.07  (0.88, 1.31) | 1.09  (0.87, 1.36) | 0.98  (0.80, 1.21) | 1.02  (0.81, 1.28) |
| **Socioeconomic position (reference group: manual)** | | | | | |
| FV | 3.21  (2.25, 4.58) | 2.08  (1.56, 2.79) | 2.10  (1.54, 2.87) | 1.76  (1.35, 2.28) | 1.60  (1.20, 2.62) |
| Salt | 0.80  (0.65, 0.98) | 0.77  (0.62, 0.96) | 0.79  (0.62, 1.01) | 1.08  (0.87, 1.35) | 1.09  (0.85, 1.39) |
| Oily fish | 1.92  (1.38, 2.66) | 2.18  (1.61, 2.94) | 2.18  (1.57, 3.03) | 2.11  (1.59, 2.79) | 1.99  (1.45, 2.73) |
| RPM | 1.27  (1.04, 1.55) | 1.43  (1.16, 1.75) | 1.46  (1.16, 1.83) | 1.16  (0.95, 1.43) | 1.21  (0.96, 1.52) |
| **Ethnicity (reference group: white)** | | | | | |
| FV | 2.66  (1.40, 5.03) | 1.93  (1.16, 3.22) | 1.79  (1.02, 3.14) | 1.90  (1.30, 2.78) | 2.18  (1.43, 3.33) |
| Salt | 4.47  (2.65, 7.54) | 3.47  (2.11, 5.72) | 3.36  (2.08, 5.43) | 1.90  (1.29, 2.80) | 2.35  (1.44, 3.83) |
| Oily fish | 0.81  (0.32, 2.06) | 1.72  (1.01, 2.91) | 1.71  (0.94, 3.11) | 1.24  (0.81, 1.90) | 1.09  (0.68, 1.75) |
| RPM | 1.87  (1.15, 3.05) | 2.03  (1.29, 3.18) | 2.08  (1.28, 3.40) | 1.80  (1.28, 2.53) | 2.06  (1.41, 3.01) |
| FV, fruit and vegetables. RPM, red and processed meat. Odds ratios are adjusted for sex, age, socioeconomic position, and ethnicity. | | | | | |
