# Supplementary material for: Time trends in adherence to UK dietary recommendations and associated sociodemographic inequalities, 1986-2012: a repeated cross-sectional analysis
Source: Eur J Clin Nutr. 2018 Nov 16;73(7):997–1005. doi: 10.1038/s41430-018-0347-z (PMC6398578; doi:10.1038/s41430-018-0347-z)
Supplement: Supplementary file 4 — Supplementary Table S2 [file 41430_2018_347_MOESM4_ESM.docx]

**Supplementary Table S2.** Weighted vs unweighted data: adjusted odds ratios (95% CIs) for adhering to dietary recommendations over time.

|  |  | **2000-01 vs 1986-87 (unweighted)**  OR (95% CI) | **2000-01 vs 1986-87 (00-01 weighted)**  OR (95% CI) | **2008-12 vs 2000-01 (unweighted)**  OR (95% CI) | **2008-12 vs 2000-01 (weighted)**  OR (95% CI) |
| --- | --- | --- | --- | --- | --- |
| **Sex** | | | | | |
| FV | Men | 1.72 (1.27, 2.32) | 1.67 (1.23, 2.27) | 1.27 (0.96, 1.67) | 1.35 (1.00, 1.82) |
|  | Women | 2.13 (1.59, 2.86) | 2.13 (1.58, 2.88) | 1.32 (1.04, 1.68) | 1.26 (0.97, 1.64) |
| Salt | Men | 1.28 (0.98, 1.67) | 1.57 (0.87, 1.54) | 3.25 (2.54, 4.14) | 3.62 (2.76, 4.76) |
|  | Women | 1.25 (1.04, 1.49) | 1.23 (1.02, 1.48) | 2.20 (1.80, 2.70) | 2.08 (1.67, 2.60) |
| OF | Men | 1.31 (0.98, 1.74) | 1.20 (0.90, 1.61) | 1.21 (0.91, 1.60) | 1.29 (0.95, 1.75) |
|  | Women | 2.47 (1.79, 3.41) | 2.42 (1.74, 3.36) | 1.30 (1.01, 1.67) | 1.32 (1.00, 1.74) |
| RPM | Men | 1.90 (1.51, 2.39) | 1.66 (1.30, 2.11) | 1.07 (0.86, 1.35) | 1.32 (1.02, 1.69) |
|  | Women | 1.70 (1.42, 2.04) | 1.66 (1.38, 2.01) | 0.77 (0.64, 0.93) | 0.78 (0.64, 0.96) |
| **Age** | | | | | |
| FV | 19-40 | 1.35 (0.95, 1.92) | 1.28 (0.89, 1.84) | 1.66 (1.20, 2.30) | 1.76 (1.23, 2.52) |
|  | 41-64 | 2.31 (1.78, 3.00) | 2.34 (1.79, 3.07) | 1.15 (0.92, 1.43) | 1.11 (0.87, 1.40) |
| Salt | 19-40 | 1.25 (1.01, 1.54) | 1.18 (0.95, 1.46) | 2.15 (1.71, 2.71) | 2.19 (1.70, 2.82) |
|  | 41-64 | 1.28 (1.04, 1.57) | 1.20 (0.98, 1.48) | 3.08 (2.49, 3.81) | 3.28 (2.59, 4.14) |
| OF | 19-40 | 1.75 (1.23, 2.49) | 1.47 (1.02, 2.12) | 1.45 (1.05, 1.99) | 1.69 (1.18, 2.42) |
|  | 41-64 | 1.72 (1.33, 2.24) | 1.75 (1.34, 2.30) | 1.17 (0.93, 1.47) | 1.10 (0.86, 1.42) |
| RPM | 19-40 | 1.76 (1.44, 2.15) | 1.63 (1.32, 2.01) | 0.93 (0.75, 1.15) | 1.05 (0.83, 1.34) |
|  | 41-64 | 1.76 (1.45, 2.15) | 1.64 (1.34, 2.00) | 0.85 (0.70, 1.03) | 0.94 (0.76, 1.16) |
| **Socioeconomic position** | | | | | |
| FV | NM | 1.67 (1.30, 2.14) | 1.63 (1.26, 2.11) | 1.24 (1.00, 1.54) | 1.20 (0.94, 1.52) |
|  | M | 2.52 (1.72, 3.71) | 2.48 (1.67, 3.68) | 1.46 (1.05, 2.02) | 1.58 (1.11, 2.25) |
| Salt | NM | 1.24 (1.01, 1.52) | 1.19 (0.97, 1.47) | 3.03 (2.47, 3.72) | 3.11 (2.49, 3.90) |
|  | M | 1.30 (1.05, 1.61) | 1.21 (0.97, 1.51) | 2.14 (1.68, 2.72) | 2.20 (1.68, 2.87) |
| OF | NM | 1.81 (1.40, 2.35) | 1.71 (1.31, 2.23) | 1.25 (1.00, 1.56) | 1.26 (0.99, 1.61) |
|  | M | 1.59 (1.11, 2.28) | 1.51 (1.04, 2.19) | 1.28 (0.90, 1.82) | 1.39 (0.95, 2.05) |
| RPM | NM | 1.86 (1.54, 2.25) | 1.72 (1.42, 2.09) | 0.82 (0.68, 0.99) | 0.93 (0.76, 1.15) |
|  | M | 1.65 (1.34, 1.99) | 1.53 (1.22, 1.91) | 0.97 (0.77, 1.22) | 1.07 (0.83, 1.37) |
| **Ethnicity** | | | | | |
| FV | White | 1.94 (1.57, 2.41) | 1.94 (1.55, 2.42) | 1.30 (1.07, 1.57) | 1.27 (1.03, 1.57) |
|  | Non-white | 1.52 (0.69, 3.34) | 1.33 (0.58, 3.05) | 1.31 (0.71, 2.41) | 1.60 (0.82, 3.14) |
| Salt | White | 1.27 (1.09, 1.48) | 1.21 (1.03, 1.41) | 2.76 (2.35, 3.25) | 2.82 (2.35, 3.37) |
|  | Non-white | 1.04 (0.54, 2.03) | 0.98 (0.51, 1.88) | 1.43 (0.80, 2.54) | 1.72 (0.92, 3.21) |
| OF | White | 1.67 (1.35, 2.07) | 1.58 (1.27, 1.98) | 1.29 (1.06, 1.57) | 1.34 (1.08, 1.66) |
|  | Non-white | 3.55 (1.24, 10.10) | 3.32 (1.09, 10.16) | 0.92 (0.48, 1.75) | 0.90 (0.44, 1.82) |
| RPM | White | 1.76 (1.52, 2.03) | 1.62 (1.40, 1.88) | 0.88 (0.76, 1.02) | 0.98 (0.83, 1.16) |
|  | Non-white | 2.03 (1.07, 3.85) | 1.90 (0.98, 3.70) | 0.82 (0.48, 1.42) | 1.00 (0.56, 1.79) |
| FV, fruit and vegetables. OF, oily fish. RPM, red and processed meat. NM, non-manual. M, manual.  Odds ratios are adjusted for sex, age, socioeconomic position, and ethnicity. | | | | | |
