# Supplementary material for: Time trends in adherence to UK dietary recommendations and associated sociodemographic inequalities, 1986-2012: a repeated cross-sectional analysis
Source: Eur J Clin Nutr. 2018 Nov 16;73(7):997–1005. doi: 10.1038/s41430-018-0347-z (PMC6398578; doi:10.1038/s41430-018-0347-z)
Supplement: Supplementary file 5 — Supplementary Table S3 [file 41430_2018_347_MOESM5_ESM.docx]

**Supplementary Table S3.** Sex inequalities: *n* (%) adhering to dietary recommendations and adjusted odds ratios (95% CIs) for adherence.

|  | | **1986-1987**  (*n*=2018)  *n* (%) | **2000-2001**  (*n*=1683)  *n* (%) | **2008-2012**  (*n*=1632)  *n* (%) | **00-01 vs 86-87**  **08-12 vs 00-01**  OR (95% CI) | **χ2**  **(*P*_interaction_)** |
| --- | --- | --- | --- | --- | --- | --- |
| FV | Men | 88 (8.9) | 119 (15.8) | 143 (20.3) | 1.72 (1.27, 2.32)  1.27 (0.96, 1.67) | 1.27  (0.53) |
|  | Women | 80 (7.8) | 152 (16.3) | 198 (21.4) | 2.13 (1.59, 2.86)  1.32 (1.04, 1.68) |  |
| OR (95% CI):  women vs men | | 0.86  (0.63, 1.19) | 1.07  (0.82, 1.40) | 1.07  (0.84, 1.37) |  | |
| Salt | Men | 138 (13.9) | 131 (17.4) | 292 (41.4) | 1.28 (0.98, 1.67)  3.25 (2.54, 4.14) | 9.30  (0.01*) |
|  | Women | 552 (53.8) | 551 (59.3) | 710 (76.6) | 1.25 (1.04, 1.49)  2.20 (1.80, 2.70) |  |
| OR (95% CI):  women vs men | | 7.59  (6.07, 9.48) | 7.18  (5.69, 9.07) | 4.76  (3.83, 5.91) |  | |
| Oily fish | Men | 110 (11.1) | 115 (15.3) | 128 (18.2) | 1.31 (0.98, 1.74)  1.21 (0.91, 1.60) | 13.76  (0.001*) |
|  | Women | 61 (5.9) | 135 (14.5) | 175 (18.9) | 2.47 (1.69, 3.41)  1.30 (1.01, 1.67) |  |
| OR (95% CI):  women vs men | | 0.50  (0.36, 0.69) | 0.95  (0.73, 1.26) | 1.05  (0.81, 1.35) |  | |
| RPM | Men | 168 (17.0) | 217 (28.8) | 222 (31.5) | 1.90 (1.51, 2.39)  1.07 (0.86, 1.35) | 11.52  (0.003*) |
|  | Women | 434 (42.3) | 522 (56.1) | 467 (50.4) | 1.70 (1.42, 2.04)  0.77 (0.64, 0.93) |  |
| OR (95% CI):  women vs men | | 3.63  (2.95, 4.47) | 3.20  (2.60, 3.93) | 2.22  (1.81, 2.73) |  | |
| FV, fruit and vegetables. RPM, red and processed meat. ******P*≤0.05. Odds ratios are adjusted for age, socioeconomic position, and ethnicity. | | | | | | |
