# Supplementary material for: Time trends in adherence to UK dietary recommendations and associated sociodemographic inequalities, 1986-2012: a repeated cross-sectional analysis
Source: Eur J Clin Nutr. 2018 Nov 16;73(7):997–1005. doi: 10.1038/s41430-018-0347-z (PMC6398578; doi:10.1038/s41430-018-0347-z)
Supplement: Supplementary file 6 — Supplementary Table S4 [file 41430_2018_347_MOESM6_ESM.docx]

**Supplementary Table S4.** Age inequalities: *n* (%) adhering to dietary recommendations and adjusted odds ratios (95% CIs) for adherence.

|  | | **1986-1987**  (*n*=2018)  *n* (%) | **2000-2001**  (*n*=1683)  *n* (%) | **2008-2012**  (*n*=1632)  *n* (%) | **00-01 vs 86-87**  **08-12 vs 00-01**  OR (95% CI) | **χ2**  **(*P*_interaction_)** |
| --- | --- | --- | --- | --- | --- | --- |
| FV | 19-40 | 67 (6.4) | 70 (8.8) | 109 (15.1) | 1.35 (0.95, 1.92)  1.66 (1.20, 2.30) | 6.58  (0.04*) |
|  | 41-64 | 101 (10.5) | 201 (22.6) | 232 (25.4) | 2.31 (1.78, 3.00)  1.15 (0.92, 1.43) |  |
| OR (95% CI):  41-64 vs 19-40 | | 1.74  (1.26, 2.42) | 2.98  (2.22, 4.00) | 2.02  (1.56, 2.62) |  | |
| Salt | 19-40 | 357 (33.8) | 319 (40.2) | 413 (57.4) | 1.25 (1.01, 1.54)  2.15 (1.71, 2.71) | 8.49  (0.01*) |
|  | 41-64 | 333 (34.6) | 363 (40.8) | 589 (64.6) | 1.28 (1.04, 1.57)  3.08 (2.49, 3.81) |  |
| OR (95% CI):  41-64 vs 19-40 | | 1.07  (0.87, 1.31) | 1.11  (0.89, 1.38) | 1.51  (1.21, 1.88) |  | |
| Oily fish | 19-40 | 60 (5.7) | 78 (9.8) | 103 (14.3) | 1.75 (1.23, 2.49)  1.45 (1.05, 1.99) | 1.62  (0.44) |
|  | 41-64 | 111 (11.5) | 172 (19.3) | 200 (21.9) | 1.72 (1.33, 2.24)  1.17 (0.93, 1.47) |  |
| OR (95% CI):  19-40 vs 41-64 | | 2.15  (1.55, 3.00) | 2.15  (1.61, 2.87) | 1.71  (1.31, 2.23) |  | |
| RPM | 19-40 | 308 (29.2) | 343 (43.2) | 310 (43.1) | 1.76 (1.44, 2.15)  0.93 (0.75, 1.15) | 0.43  (0.81) |
|  | 41-64 | 294 (30.5) | 396 (44.5) | 379 (41.6) | 1.76 (1.45, 2.15)  0.85 (0.70, 1.03) |  |
| OR (95% CI):  41-64 vs 19-40 | | 1.07  (0.87, 1.30) | 1.07  (0.88, 1.31) | 0.98  (0.80, 1.21) |  | |
| FV, fruit and vegetables. RPM, red and processed meat. ******P*≤0.05. Odds ratios are adjusted for sex, socioeconomic position, and ethnicity. | | | | | | |
