# Supplementary material for: Time trends in adherence to UK dietary recommendations and associated sociodemographic inequalities, 1986-2012: a repeated cross-sectional analysis
Source: Eur J Clin Nutr. 2018 Nov 16;73(7):997–1005. doi: 10.1038/s41430-018-0347-z (PMC6398578; doi:10.1038/s41430-018-0347-z)
Supplement: Supplementary file 7 — Supplementary Table S5 [file 41430_2018_347_MOESM7_ESM.docx]

**Supplementary Table S5.** Socioeconomic inequalities: *n* (%) adhering to dietary recommendations and adjusted odds ratios (95% CIs) for adherence.

|  | | **1986-1987**  (*n*=2018)  *n* (%) | **2000-2001**  (*n*=1683)  *n* (%) | **2008-2012**  (*n*=1632)  *n* (%) | **00-01 vs 86-87**  **08-12 vs 00-01**  OR (95% CI) | **χ2**  **(*P*_interaction_)** |
| --- | --- | --- | --- | --- | --- | --- |
| FV | Non-manual | 123 (12.6) | 196 (20.2) | 240 (24.3) | 1.67 (1.30, 2.14)  1.24 (1.00, 1.54) | 7.14  (0.03*) |
|  | Manual | 45 (4.3) | 75 (10.5) | 101 (15.7) | 2.52 (1.72, 3.71)  1.46 (1.05, 2.02) |  |
| OR (95% CI):  NM vs M | | 3.21  (2.25, 4.58) | 2.08  (1.56, 2.79) | 1.76  (1.35, 2.28) |  | |
| Salt | Non-manual | 311 (32.0) | 374 (38.6) | 613 (62.1) | 1.24 (1.01, 1.52)  3.03 (2.47, 3.72) | 5.82  (0.05*) |
|  | Manual | 379 (36.3) | 308 (43.2) | 389 (60.3) | 1.30 (1.05, 1.61)  2.14 (1.68, 2.72) |  |
| OR (95% CI):  NM vs M | | 0.80  (0.65, 0.98) | 0.77  (0.62, 0.96) | 1.08  (0.87, 1.35) |  | |
| Oily fish | Non-manual | 108 (11.1) | 183 (18.9) | 224 (22.7) | 1.81 (1.40, 2.35)  1.25 (1.00, 1.56) | 0.36  (0.84) |
|  | Manual | 63 (6.0) | 67 (9.4) | 79 (12.2) | 1.59 (1.11, 2.28)  1.28 (0.90, 1.82) |  |
| OR (95% CI):  NM vs M | | 1.92  (1.38, 2.66) | 2.18  (1.61, 2.94) | 2.11  (1.59, 2.79) |  | |
| RPM | Non-manual | 313 (32.2) | 460 (47.4) | 430 (43.6) | 1.67 (1.30, 2.14)  0.82 (0.68, 0.99) | 1.82  (0.40) |
|  | Manual | 289 (27.7) | 279 (39.1) | 259 (40.2) | 2.52 (1.72, 3.71)  0.97 (0.77, 1.22) |  |
| OR (95% CI):  NM vs M | | 1.27  (1.04, 1.55) | 1.43  (1.16, 1.75) | 1.16  (0.95, 1.43) |  | |
| FV, fruit and vegetables. RPM, red and processed meat. NM, non-manual. M, manual.  ******P*≤0.05. Odds ratios are adjusted for sex, age, and ethnicity. | | | | | | |
