# Supplementary material for: Time trends in adherence to UK dietary recommendations and associated sociodemographic inequalities, 1986-2012: a repeated cross-sectional analysis
Source: Eur J Clin Nutr. 2018 Nov 16;73(7):997–1005. doi: 10.1038/s41430-018-0347-z (PMC6398578; doi:10.1038/s41430-018-0347-z)
Supplement: Supplementary file 8 — Supplementary Table S6 [file 41430_2018_347_MOESM8_ESM.docx]

**Supplementary Table S6.** Ethnic inequalities: *n* (%) adhering to dietary recommendations and adjusted odds ratios (95% CIs) for adherence.

|  | | **1986-1987**  (*n*=2018)  *n* (%) | **2000-2001**  (*n*=1683)  *n* (%) | **2008-2012**  (*n*=1632)  *n* (%) | **86-87 vs 00-01**  **86-87 vs 08-12**  OR (95% CI) | **χ2**  **(*P*_interaction_)** |
| --- | --- | --- | --- | --- | --- | --- |
| FV | White | 155 (8.0) | 248 (15.6) | 296 (20.1) | 1.94 (1.57, 2.41)  1.30 (1.07, 1.57) | 0.85  (0.65) |
|  | Non-  White | 13 (16.7) | 23 (25.6) | 45 (28.3) | 1.52 (0.69, 3.34)  1.31 (0.71, 2.41) |  |
| OR (95% CI): NW vs W | | 2.66  (1.40, 5.03) | 1.93  (1.16, 3.22) | 1.90  (1.30, 2.78) |  | |
| Salt | White | 643 (33.1) | 624 (39.2) | 889 (60.4) | 1.27 (1.09, 1.48)  2.76 (2.35, 3.25) | 7.52  (0.02*) |
|  | Non-  White | 47 (60.3) | 58 (64.4) | 113 (71.1) | 1.04 (0.54, 2.03)  1.43 (0.80, 2.54) |  |
| OR (95% CI): NW vs W | | 4.47  (2.65, 7.54) | 3.47  (2.11, 5.72) | 1.90  (1.29, 2.80) |  | |
| Oily fish | White | 166 (8.6) | 230 (14.4) | 272 (18.5) | 1.94 (1.57, 2.41)  1.29 (1.06, 1.57) | 2.11  (0.35) |
|  | Non-  White | 5 (6.4) | 20 (22.2) | 31 (19.5) | 1.32 (0.69, 3.34)  0.92 (0.48, 1.75) |  |
| OR (95% CI): NW vs W | | 0.81  (0.32, 2.06) | 1.72  (1.01, 2.91) | 1.24  (0.81, 1.90) |  |  |
| RPM | White | 570 (29.4) | 685 (43.0) | 602 (40.9) | 1.76 (1.52, 2.03)  0.88 (0.76, 1.02) | 0.11  (0.95) |
|  | Non-  White | 32 (41.0) | 54 (60.0) | 87 (54.7) | 2.03 (1.07, 3.85)  0.82 (0.48, 1.42) |  |
| OR (95% CI): NW vs W | | 1.87  (1.15, 3.05) | 2.03  (1.29, 3.18) | 1.80  (1.28, 2.53) |  | |
| FV, fruit and vegetables. RPM, red and processed meat. NW, non-white. W, white.  ******P*≤0.05.  Odds ratios are adjusted for sex, age, and socioeconomic position. | | | | | | |
