# Supplementary material for: Time trends in adherence to UK dietary recommendations and associated sociodemographic inequalities, 1986-2012: a repeated cross-sectional analysis
Source: Eur J Clin Nutr. 2018 Nov 16;73(7):997–1005. doi: 10.1038/s41430-018-0347-z (PMC6398578; doi:10.1038/s41430-018-0347-z)
Supplement: Supplementary file 9 — Supplementary Table S7 [file 41430_2018_347_MOESM9_ESM.docx]

**Supplementary Table S7.** Sociodemographic inequalities in meeting dietary recommendations for fruit and vegetables, oily fish and red and processed meat, comparing non-disaggregated and disaggregated data from NDNS Rolling Programme (2008-2012).

|  | **Fruit and vegetables** | | **Oily fish** | | **Red and processed meat** | |
| --- | --- | --- | --- | --- | --- | --- |
|  | Non-disaggregated | Disaggregated | Non-disaggregated | Disaggregated | Non-disaggregated | Disaggregated |
| Overall | 20.9 | 30.4 | 18.6 | 16.2 | 42.2 | 62.8 |
| Men, % | 20.3 | 28.9 | 18.2 | 16.6 | 31.5 | 47.8 |
| Women, % | 21.4 | 31.5 | 18.9 | 15.9 | 50.4 | 74.2 |
| Women vs men, OR (95% CI) | 1.07 (0.84, 1.37) | 1.13 (0.91, 1.41) | 1.05 (0.81, 1.35) | 0.94 (0.72, 1.23) | 2.22 (1.81, 2.73) | 3.19 (2.59, 3.94) |
| 19-40 years, % | 15.1 | 23.9 | 14.3 | 12.1 | 43.1 | 63.8 |
| 41-64 years, % | 25.4 | 35.5 | 21.9 | 19.4 | 41.6 | 62.1 |
| 41-64 years vs 19-40 years, OR (95% CI) | 2.02 (1.56, 2.62) | 1.84 (1.46, 2.30) | 1.71 (1.31, 2.23) | 1.77 (1.33, 2.34) | 0.98 (0.80, 1.21) | 0.98 (0.79, 1.21) |
| Non-manual, % | 24.3 | 35.1 | 22.7 | 19.7 | 43.6 | 65.8 |
| Manual, % | 15.7 | 23.3 | 12.3 | 10.9 | 40.2 | 58.3 |
| Non-manual vs manual, OR (95% CI) | 1.76 (1.35, 2.28) | 1.81 (1.43, 2.27) | 2.11 (1.59, 2.79) | 2.01 (1.50, 2.70) | 1.16 (0.95, 1.43) | 1.42 (1.15, 1.76) |
| White, % | 20.1 | 29.6 | 18.5 | 16.2 | 40.9 | 61.4 |
| Non-white, % | 28.3 | 37.7 | 19.5 | 16.4 | 54.7 | 75.5 |
| Non-white vs white, OR (95% CI) | 1.90 (1.30, 2.78) | 1.71 (1.21, 2.43) | 1.24 (0.81, 1.90) | 1.18 (0.75, 1.86) | 1.80 (1.28, 2.53) | 2.07 (1.40, 3.07) |
| Odds ratios are adjusted for sex, age, socioeconomic position, and ethnicity. | | | | | | |

Non-disaggregated and disaggregated average daily intake estimates were available for respondents in the NDNS Rolling Programme. Non-disaggregated estimates code 100% of a mixed dish by its meat/fish component, e.g. lamb stew is coded as 100% lamb. Disaggregated estimates separate a mixed dish into its individual ingredients, e.g. the same lamb stew could be 60% lamb, 10% onions, 5% carrots etc. We compared adherence to dietary recommendations for fruit and vegetables, oily fish and red and processed meat using both methods of intake estimation, overall and across sociodemographic subgroups. The methods used for disaggregated data was the same as the methods used for non-disaggregated data presented in the main report. Overall adherence was 10% higher for fruit and vegetables, 2% lower for oily fish, and 20% higher for red and processed meat, when using disaggregated estimates compared to non-disaggregated estimates. The inequalities observed were similar between the two intake estimation methods for the fruit and vegetables and oily fish recommendations. For red and processed meat, was saw greater sex and socioeconomic inequalities when using disaggregated data compared to non-disaggregated data, but similar odds ratios for age and ethnic inequalities.
